# Why do women not return for CD4 count results at Embhuleni Hospital, Mpumalanga, South Africa?

**DOI:** 10.4102/curationis.v38i1.1266

**Published:** 2015-06-26

**Authors:** Doudou K. Nzaumvila, Langalibalele H. Mabuza

**Affiliations:** 1 Family Physician, Odi District Hospital, South Africa; 2 Department of Family Medicine and Primary Health Care, University of Limpopo, Medunsa Campus, South Africa

## Abstract

**Background:**

According to the South African *Policy and guidelines for the implementation of the PMTCT programme* of 2008, all pregnant women who tested HIV-positive also had to have their CD4 count measured in order to inform the option of Prevention of Mother-to-Child Treatment (PMTCT): to be put on lifelong treatment or to be placed on temporary PMTCT. They were required to return for the results within two weeks, but some did not return, implying that they did not benefit from the programme. This study was conducted to establish their reasons for not returning.

**Objectives:**

To explore the reasons given by women attending antenatal care for not returning for the results of their CD4 count done for PMTCT at Embhuleni Hospital and satellite clinics, Mpumalanga.

**Methods:**

The study was a qualitative study using the free-attitude interview technique. Women who had not returned for their results were traced and interviewed on their reasons for not returning. Interviews were conducted in Siswati, audio-taped, transcribed verbatim and translated into English for analysis. Data saturation was reached by the eighth participant. A thematic analysis was conducted.

**Results:**

The themes that emerged were: participants were not informed about the PMTCT process; poor service delivery from the healthcare practitioners; unprofessional healthcare practitioners’ conduct; shortages of medication in the healthcare facilities; fear of social stigma; and poor patient socioeconomic conditions.

**Conclusion:**

The reasons for not returning were mainly based on participants’ experiences during consultations at the healthcare centres and their perceptions of the healthcare practitioners. Healthcare practitioners should adhere to the tenets of professionalism in order to address this problem.

## Introduction

By the turn of the century, the HIV pandemic had become a global problem, especially in sub-Saharan Africa. By 2006, 25 million adults and children were reported to be living with the virus in this region (Draper & Abdullah 2004:431; UNAIDS/World Health Organization [WHO] 2006:3). At that time, more than four million children were estimated to have died from HIV-related disease, primarily as a result of mother-to-child transmission (MTCT) of HIV. MTCT of HIV remains the most common route of HIV infection for HIV-positive children under the age of five years, with the virus being transmitted either pre- or perinatally (during pregnancy, labour or delivery), or postnatally, by means of breastfeeding (O’ Gorman, Nyirenda & Theobald 2010:2; WHO 2013:27–31). To meet this challenge, the programme of prevention of mother-to-child transmission (PMTCT) was introduced by the United Nations Children’s Fund (UNICEF) in 2001 in Mali, to protect children from contracting HIV from their mothers. The programme, described by UNICEF as ‘the most effective way to create an HIV-free generation’ (UNICEF 2001:13), has since been adopted globally. Voluntary Counselling and Testing (VCT) was provided to the pregnant women, followed by free treatment for HIV for those who tested positive for the virus (UNICEF 2010:17).

Since 2001, there has been an improvement in the intensity of the treatment of pregnant women living with the virus. Initially, they received only Nevirapine (NVP) during labour, which was followed by the introduction of dual therapy (NVP and Zidovudine [azidothymidine, or AZT]), with the ultimate aim of introducing triple therapy for PMTCT at all sites worldwide by 2007. However, as late as 2010, this ideal had not yet been realised on a global scale (UNICEF 2010:17):

In 2009, an estimated 860,000 pregnant women were found to be living with HIV in Eastern and Southern Africa, more than in any other region of the world. The region is also home to 47 percent of the global total of children living with HIV, of which over 90 percent were infected through vertical transmission from the mother to the baby during pregnancy, delivery or breastfeeding. (UNICEF 2005)

According to the South African *Policy and guidelines for the implementation of the PMTCT programme* of 2008 (National Department of Health 2008), all pregnant women attending antenatal care (ANC) were to be managed according to the four stages of PMTCT: (1) primary prevention of HIV; (2) antenatal; (3) labour and delivery; and (4) postnatal. The first stage comprised primary prevention of HIV and MTCT with the goal of reducing prevalence of HIV amongst women of child-bearing age. The second stage focused on ANC with the goal of increasing management coverage of HIV-positive pregnant women. The objectives of this stage were, *inter alia*, to: identify the pregnant women who were HIV-positive; ensure that the HIV-positive pregnant women enrolled in the PMTCT programme; do a CD4 count on all HIV-positive pregnant women; assess them according to the WHO clinical staging guidelines; encourage repeat HIV testing not later than 34 weeks during pregnancy; and prescribe AZT from 28 weeks’ gestation for those who had a CD4 count ≥ 200 cells/mL, or lifelong highly-active antiretroviral therapy (HAART) for those with a CD4 count < 200 cells/mL and who were at WHO stages 3 and 4. The third stage focused on labour and delivery, with the goal of minimising the risk of MTCT of HIV during this stage. The fourth and last stage, which was not the focus of this study, was aimed at reducing the risk of postnatal transmission of HIV through postnatal follow-up of both mother and infant (National Department of Health 2008:16–19).

At the time of the study, in accordance with the 2008 Guidelines (National Department of Health 2008), all pregnant women attending ANC at the Embhuleni Hospital and its satellite clinics received VCT for HIV. A rapid HIV test was done on a drop of blood obtained through a finger prick. If the rapid HIV test was positive, a confirmatory rapid HIV test was done utilising a different brand of rapid HIV test. The healthcare practitioner had to ensure that the initial and the confirmatory tests were done in the presence of the woman being tested. A woman was considered HIV-positive if the second rapid test was also positive. She was then given her results and post-test counselled. If the woman tested positive for HIV, blood was then taken for a CD4 count so as to inform appropriate management of the patient throughout pregnancy and delivery (National Department of Health 2008:16). The CD4 count results were available within two weeks of the bloods being drawn. Those who tested HIV negative were also counselled according to protocol and offered a repeat HIV test at or around 34 weeks so as to detect late sero-converters.

The South African antiretroviral treatment guidelines were updated in 2010 and then again in 2013. According to the *South African antiretroviral treatment guidelines 2010* (National Department of Health 2010), a pregnant woman was eligible for ART with a CD4 count of 350 cells/mL and below, whereupon she had to be started on lifelong treatment with Tenofovir (TDF) plus either Lamivudine (3TC) or Emtricitabine (FTC) plus NVP within two weeks of her pregnancy. The *South African antiretroviral treatment guidelines 2013* (National Department of Health 2013:6) introduced the fixed drug combination (FDC) which is given to all pregnant women at their first antenatal visit (any gestational stage). On their second visit, which is a week later, their serum creatinine level and CD4 count are monitored. A woman with a serum creatinine of 85 µmol/L, irrespective of their CD4 count, will continue with the FDC, whilst those with a serum creatinine above 85 µmol/L will be given AZT instead of TDF because of the nephrotoxic effect of the latter. In both of these updated guidelines, the CD4 count remains an important guiding factor in the management of the pregnant woman, highlighting the compelling need for her return for the results and further management at her next appointment date.

At the time of the study, the principal researcher observed from the antenatal clinic and the labour ward hospital records that of all the pregnant women who had tested positive for HIV, only about one in four would return for their CD4 result. The rest would only present later with subsequent pregnancies or complications of HIV, such as opportunistic infections. This study aimed to explore the reasons for the non-return, with the hope that it would enable policy makers for Embhuleni Hospital and its catchment areas to devise informed policies to meet the challenge.

### Significance of the study

The significance of the study lies in the identification of reasons for women who had not returned for the results their CD4 tests. It is hoped that establishing these reasons, which could be stumbling blocks encountered by patients, will enable health workers to address them in an effort to improve healthcare. Some of the reasons may not pertain directly to the health fraternity, necessitating collaboration with other relevant bodies. Policies can then be formulated toward achievement of comprehensive mother and child health.

### Problem statement

Embhuleni Hospital is located in the rural Elukwatini District in the Albert Luthuli local municipality in the Mpumalanga Province of South Africa. It is near the eastern border of Swaziland. At the time of the study (2008), the 220-bed hospital served 15 satellite clinics, 3 community health centres and 2 mobile clinics. At that time, hospital records estimated that only 1 in 10 of the patients in these healthcare facilities were women attending ANC. The principal researcher noticed from the hospital labour ward records that only about a quarter of the women who had tested positive for HIV returned for their CD4 count results. Their return for these results was important in the proper implementation of the PMTCT programme for each affected woman. The study was conducted to establish their reasons for not returning.

### Research question

The research question of this article was: ‘What reasons did the women who had attended ANC at Embhuleni Hospital and the satellite clinics give for not returning for their CD4 count results for PMTCT?’

## Research methods and design

### Design

The study was a qualitative study using the free-attitude interview technique to explore the phenomenon (Vrolijk 2007:52–76; Willig 2001:30).

### Population

The study population comprised all the women 18 years and above who had visited an ANC clinic, received VCT and had tested HIV-positive, but had not returned for their CD4 count results.

The principal researcher traced eligible participants from the hospital labour ward records. All the women whose record indicated that they had visited an ANC clinic, received VCT, had tested HIV-positive, but had not returned for their CD4 count results were requested to participate. He phoned the eligible participants and made an appointment to visit them in their home so as to explain the study and recruit them for participation. Those who consented to participate signed the informed consent. The study excluded participants who could not communicate in the local language, Siswati; those who tested HIV negative; those who were already on lifelong HIV treatment; as well as those who, on arrival, were already in labour.

### Data collection methods

Data collection was done in a selected secluded room at Embhuleni Hospital on 20 June 2009. The free-attitude interview technique was used (Vrolijk 2007:52–76; Willig 2001:30). This technique is described as a non-directive controlled interview. The research assistant asked each participant the same exploratory question in Siswati: *Ngicela ungiphe tizatfu letente wangaseti kutewuva imiphumela yekuhlolwa kwelizinga lemasotja emtimba wakho engatini leyatsatfwa lesikhatsi usatetfwele?* [Would you please give me the reason for not coming back for your CD4 count results following the test during your pregnancy?]. No other questions were asked, except for clarification questions to ensure accurate understanding of the issues raised by the participant. The research assistant offered reflective summaries at the conclusion of each idea under discussion for verification by the participant. All the interviews were tape-recorded and field notes were taken. Field notes included the participant’s non-verbal expressions: for example, in a case where a participant expressed anger through facial expression, such body language would be recorded. Each interview took about 45 minutes to one hour to complete. The interviews continued until, by the eighth interview, there was no new information gathered (data saturation).

### Data analysis

A thematic data analysis approach was used (Lincoln & Guba 1985:18). The researchers immersed themselves in the participants’ responses so as to familiarise themselves with the presented data. The data were coded and grouped into categories. Group consensus on themes was achieved at a group meeting comprising the three researchers. The identified themes were subjected to researcher triangulation by the research team – the team subjected data to an iterative process, whereby the data were visited and revisited, connecting them with emerging insights and progressively refining the focus of understanding (Srivastava & Hopwood 2009). Member checking to authenticate the study findings through eliciting feedback from each participant was done (Riessman 2008:38).

## Results

Eight participants were interviewed. Their ages ranged from 18 to 39 and all but one were unemployed. They resided in the rural area in the neighbourhood of the hospital, as described above. Their academic level ranged from none to secondary education and all were single, with parity ranging from one to five children (Table 1).

**TABLE 1:** Themes on reasons for not returning for CD4 count results.

Theme	Topic
Theme 1:	Not informed about the PMTCT process.
Theme 2:	Poor service rendered by the healthcare practitioners.
Theme 3:	Unprofessional conduct of healthcare practitioners.
Theme 4:	Shortage of medication at the healthcare facility.
Theme 5:	Fear of social stigma.
Theme 6:	Poor patient socioeconomic conditions.

PMTCT, prevention of mother-to-child transmission of HIV.

### Theme 1: Not informed about the prevention of mother-to-child transmission process

The participants stated that they were not made aware that they had to come back for the CD4 count results. The whole PMTCT process was not made clear to them in terms of what was expected of them and the underlying reasons:

‘I didn’t know I had to come after having the baby … [*t*]hat is the reason I ended up staying at home.’ (KS, 26, unemployed, three children)

‘They told me nothing, they just filled my blood in some containers that looked purple and red and they told me next time I come back, they will give me some white pills…’ (NM, 18, unemployed, one child)

‘They told me I would get pills when I became really sick … and said I had delayed coming to the clinic as I only came when I was seven or eight months pregnant … they never told me to come back, so I stayed at home.’ (KS, 26, unemployed, three children)

‘At the clinic they say nothing … they just teach us about AIDS, that as a person you must know your status. They said I needed to take my pills and take them when my CD4 count was down. I didn’t know that I had to come back … well the nurses didn’t tell me.’ (ND, 18, unemployed, one child)

Some thought that the test was done for statistical purposes only:

‘I thought maybe it’s the way they do things, just to discover how many of the people are like this, and at the end of the day, they just … count numbers and that’s it.’ (PZ, 38, employed, two children)

However, one participant, when asked what she had been told after blood was drawn from her for a test, initially indicated that she had forgotten what she had been told. Then she suddenly remembered and conceded that she had actually been told to come back for her CD4 count results:

‘I don’t remember. They told me I must check my CD count … to see how strong they are … they told me to come back.’ (DM, 28, unemployed, one child)

Others indicated that they thought they would be contacted by the clinic personnel when the results came back:

‘After the blood test I was expecting them to call me, but nothing happened after that, no-one made any follow-up, like maybe to tell me the results are back. I just thought when the results are back they’ll call, because I don’t live far from the clinic.’ (PZ, 38, employed, two children)

### Theme 2: Poor service rendered by the healthcare practitioners

The participants indicated that the service they had received from the staff at the healthcare facilities was poor. What participants regarded as poor service comprised poor administration and being improperly addressed by the healthcare practitioners. These encounters had influenced their decision not to return:

‘On a Tuesday I came and they told me they were busy. I came back on Wednesday and again they told me to come back on another day and then I never did.’ (DM, 28, unemployed, one child)

‘When I had my baby I was so discouraged that he wasn’t given ARVs by this other nurse [*post-delivery*], so I think that’s the reason why I never came back.’ (NN, 30, unemployed, two children)

In another incident, the poor service rendered by the healthcare practitioners manifested itself through lack of comprehensive care. A mother who had brought her child at six weeks for immunisation was expecting to be given her CD4 count results as well. Although she could not explain to the interviewer the reason for not asking the nurse in attendance about her results, she expressed unhappiness at not being given her CD4 count results in addition to the care for her baby:

‘The next time I went to the clinic was for my baby’s immunisation when he was six weeks old. They gave him an injection. Nothing was said about my [*CD4 count*] results. That’s when I gave up and decided never to come back.’ (NS, 36, unemployed, five children)

### Theme 3: Unprofessional conduct of healthcare practitioners

Some participants stated that there was lack of respect for patients and no confidentiality of their conditions at the healthcare facilities:

‘… Like, I come here and talk with one of the nurses, but after that I hear the very same nurse talking with other patients about my status … if I have STIs, the nurse will just come out shouting for all to hear, telling them … you know, that they must go and get condoms. Sometimes a nurse will be heard shouting: “my office smells as if there’s dead fish lying around”. So these are the kind of things that we experience here at the clinic.’ (NN, 30, unemployed, two children)

One of the participants expressed regret at having done the test at her local clinic, as she felt there was no privacy about her status following the test. This event made her decide never to go back to that clinic:

‘I feel I made a mistake by doing my test here locally because, you will understand that … people who work with these kind of things, they don’t swear to secrecy, they talk openly after they have discovered that people are infected … so that’s one of the things that made me to stay at home. I just told myself I’m not going to go through this again … since I’m now employed, I have a medical aid, I’ve been thinking a lot about going to a private doctor, you see … but not around here. Because people talk.’ (PZ, 38, employed, two children)

Some participants had experienced unpleasant treatment from the practitioners at the healthcare facilities on previous visits. Some had been admitted to hospital and experienced neglect with regard to the fact that the healthcare practitioners did not give them their medication at the right time:

‘… I was about to be discharged, that’s when they realised that the baby needed medication … you get people who are negligent, people who are working here. They don’t follow procedures, like checking your records [*referring to records in the patient’s file*] …’ (NN, 30, unemployed, two children)

Others felt they were addressed in an improper manner by the healthcare practitioners:

‘The nurse said to me: “Why are you worried that your baby might be positive, why? She will live, so why should you worry that your baby didn’t get ARVs, she might be positive – it’s not a problem; she’ll live”. This was followed by, “shut up, you don’t know anything”. I then decided to report this sister to her supervisor in the labour ward … you get harassed; they [*referring to nurses*] speak to you as if you are a child.’ (NN, 30, unemployed, two children)

‘… [*P*]eople [referring to the nurses] are desperate for jobs, they just want some quick money … they don’t have hearts, they just shout at people [*referring to patients*] all the time.’ (PZ, 38, employed, two children)

A bad encounter with a staff member caused a participant not to go back to the same healthcare facility:

‘I was supposed to go back when my baby was six months … but was scared that I’ll see the nurse who did that to my baby because I had reported her …’ (NN, 30, unemployed, two children)

### Theme 4: Shortage of medication at the healthcare facilities

Participants had observed the general shortage of medication at their local clinics. This discouraged them with regard to coming back since they had past experiences of being told that there was no medication. They were reluctant to travel to the clinic for nothing.

‘… [*T*]hey were telling us we must come back … but it is known in the community that the clinics run out of stock with medication … sometimes when you get there you find that there are no pills at all …’ (DM, 28, unemployed, one child)

### Theme 5: Fear of social stigma

Participants expressed fear that people would get to know about their status and stigmatise them. Given the lack of privacy participants expressed with regard to the healthcare facility staff, they feared that coming back would render them vulnerable to the exposure of their status making them vulnerable to social stigma:

‘My understanding has to do with being afraid … afraid to talk about HIV, because I’m afraid people will know I’m HIV positive. I shall then be viewed in a bad light by my community.’ (NN, 30, unemployed, two children)

### Theme 6: Poor patient socioeconomic conditions

Some participants expressed a lack of financial resources for accessing food. Their comments indicated, however, that they were aware of the importance of proper nutrition when taking medication:

‘… I wished to go back so as to know the stage [*reference to CD4+ count*] where I am, so that I could start with ARVs, but my other problem was that I didn’t have enough food at home, thinking of not having enough food, while at the same time I had to drink my pills frustrated me.’ (NN, 30, unemployed, two children)

Others cited a lack of finance for transport as being responsible for their difficulty in accessing the healthcare facilities:

‘I’m poor, and I can’t afford to travel by taxi … and reaching the clinic on foot is also difficult as I have to wake up early in the morning so as to be first on the queue. After the consultation, I will have to go back home still on foot. Where I live it’s too far from the clinic.’ (SN, 39, unemployed, two children)

**FIGURE 1:**
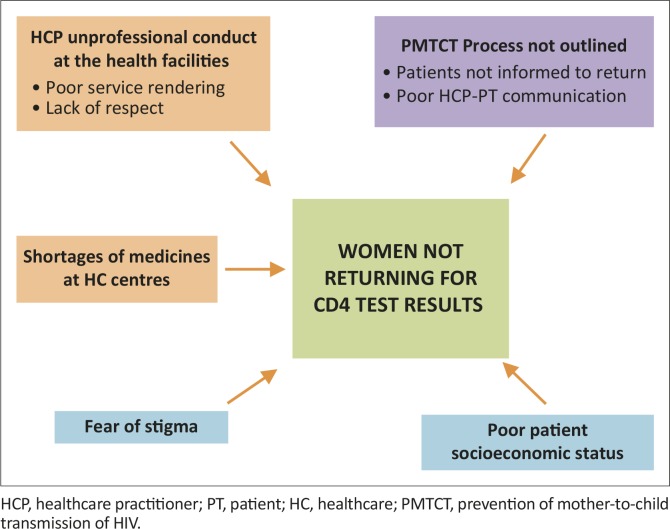
Integrated scheme – reasons for women not returning for CD4 count results. HCP, healthcare practitioner; PT, patient; HC, healthcare; PMTCT, prevention of mother-to-child transmission of HIV.

In summary, the reasons given by women for not returning for their CD4 count results for PMTCT were based mainly on participants’ experiences during consultations at the healthcare centres as well as their perceptions of the healthcare practitioners who were mainly nurses. The participants reported that they had not been informed about what the PMTCT process entailed, the service rendered at the healthcare facilities was poor, healthcare practitioners were conducting themselves unprofessionally and there was shortage of medication in the healthcare facilities. Participants lacked financial resources and they had the fear of social stigma regarding their HIV status (Figure 1).

## Ethical considerations

### Potential benefits and hazards

The research assistant conducting the interviews was a social scientist trained in qualitative research methods. The principal researcher who had recruited the participants introduced the research assistant as part of the research team to each participant before the interviews began. The research assistants explained to the participants that there were no potential risks as their participation would be kept anonymous. The potential benefits would be improved patient care through the understanding of the reasons women gave for not returning for their CD4 count results when they attended ANC for PMTCT. This understanding would enhance patient care as the healthcare facility personnel, hospital and district policy-makers would factor them in during the PMTCT programme review.

### Ethical clearance

Ethical clearance was obtained from the Medunsa Research and Ethics Committee (MREC) of the University of Limpopo – Medunsa Campus (Clearance Certificate number MREC/M/130/2008:PG). Permission to conduct the study at the hospital and the district clinics was obtained from the Superintendent of Embhuleni Hospital and the District Primary Health Care Manager, respectively. Participation in the study was voluntary.

### Informed consent

Participants were assured of confidentiality and patient respect during the study and that they were free to withdraw from participation or answering further questions, even when they had already given consent – no questions would be asked. All consenting participants signed a written consent form as recommended in Boeije (2010:30–45).

## Trustworthiness

To ensure trustworthiness of the study findings, the principles of credibility, dependability, confirmability and transferability were followed (Anfara, Brown & Mangione 2002:32). The verbatim transcription, translation of the scripts from Siswati to English and the reverse translation of the scripts back to Siswati were done in order to ensure data credibility. Furthermore, an independent senior researcher conducted a peer-review of the categories and themes acting as a ‘devil’s advocate’, challenging the research team to provide evidence for their interpretation and conclusions drawn from the data (Darbyshire, MacDougall & Schiller 2005:423). Dependability was ensured by including each participant’s response in the grouping of data categories into themes. However, the researchers looked at the range of the participants’ responses (variability) in the interest of the depth of information, rather than their average responses (Guba 1981:76). Confirmability was ensured by the non-involvement of the principal researcher in the interviews as this could influence the participants’ responses. Furthermore, field notes on the process of data collection, participants’ non-verbal expressions as well as the transcripts were used for data triangulation (Srivastava & Hopwood 2009:78). Transferability was ensured by providing a thick description of the study so as to allow evaluation regarding how well the study’s conclusions can be applied to other, similar, settings (Slade & Hoppmann 2011:67–68).

## Discussion

Participants indicated that they were not informed that they had to return for their results, suggesting that there was a problem in the healthcare practitioner-patient communication. Patients who receive poor communication from their healthcare practitioners have been found to have a 19% higher risk of non-adherence to treatment, compared with those who were communicated to effectively (Zolnierek & DiMatteo 2009:30). Although participants indicated that the nursing personnel did not make them aware they had to return for the results, it should be noted that the patients could also have forgotten what they had been told. Indeed, one participant in our study conceded that she had forgotten. This implies that the healthcare practitioner needs to ensure that patients remember the key points in the message imparted, for example, specifying the return date. Recently, some studies have explored the feasibility of using short message service (SMS) on mobile devices to improve patient attendance and have found it to be effective (Hasvold & Wootton 2011:361; Prasad & Anand 2012:24). However, a study conducted on SMS patient reminders in a rural area in Swaziland (similar to our study setting) did not find any difference between the experimental and the control groups (Kliner *et al.* 2013:4). Nevertheless, this method could be considered in the future for the hospital and its catchment clinics.

The ‘Batho Pele’ (Putting People First) Principles introduced by the South African government in 1997 were intended to instil the culture of accountability in public servants of all sectors of society (South African Government 2007:6). In the health sector, the healthcare practitioners’ attitude toward patients has been found to play a significant role in determining patient satisfaction in the healthcare practitioner-patient relationship (Loxterkamp 2013:575). In a study involving inner city London and rural Essex settings, patients who experienced poor patient-staff relations were shown to avoid returning to the same healthcare centre (Martin, Perfect & Mantle 2005). In our study, some participants raised the issue of their reluctance to return to the same healthcare facility where they had met nurses who had not treated them professionally. It stands to reason that healthcare practitioners have the responsibility to maintain professionalism at all times in order to enhance good healthcare practitioner-patient relationships. The fact that one participant stated that she had taken her baby for immunisation at six weeks and was not told of her CD4 count results reflected a missed opportunity on the part of the healthcare practitioner to explore patient’s expectations (McWhinney 1997:136). Not all patients volunteer their expectations to the healthcare practitioner. Therefore, it is upon the practitioner to make enquiries regarding other health-related matters – over and above the patient’s presenting complaint (Stott & Davis 1979:203).

Confidentiality as a component of patient autonomy is known to influence the healthcare practitioner–patient relationship (Matlakala & Mokoena 2011:484). The importance of confidentiality dates back to the Hippocratic and Florence Nightingale eras (Pinsky 2012:298–299; Wagner & Whaite 2010:229) and should be part and parcel of the healthcare practitioner’s etiquette and professionalism. In our study, participants expressed resentment regarding the lack of confidentiality about patients’ conditions which was evident in the clinics.

Unpleasant experiences have been reported by patients in health centres worldwide (Kahabuka *et al.* 2012:4–5; Tackett *et al.* 2013:910–913). These experiences have been reported by patients as ‘having lost confidence’, ‘feeling like a nuisance’ and ‘feeling abandoned and lonely’ (Jangland *et al.* 2011:432; Ridd *et al.* 2009). In our study, the loss of confidence in the healthcare practitioner (poor service rendering) and the healthcare system (shortages of medication at the healthcare facilities) could have led to the non-return on the part of the participants.

Shortage of medicines and equipment has been reported at a number of public health centres in South Africa, usually ascribed to problems in the procurement process (including the tender process), leading to non-delivery to intended destinations (Public Protector South Africa 2013). The remark by participants indicated that healthcare administrators should make an effort to identify and address the bottlenecks on healthcare delivery so as to ensure a smooth-running supply chain which will boost patient confidence on resource availability.

South Africa has a bad history of violence based on social stigma against people living with HIV. In 1998, Ms Gugu Dlamini, a 36-year-old volunteer field worker for the National Association of People Living With HIV and/or AIDS was beaten to death by her community in Durban, accusing her of bringing shame on their community by revealing her status (McNeil 1998:1). In our study, the participants had already observed in their first encounter that nurses did not respect confidentiality, so they avoided returning to the health centre for their results so as to avoid the possibility of being addressed publicly regarding their HIV status. A number of studies have indicated that social stigma about being HIV-positive have a negative effect on treatment adherence (Katz *et al*. 2013:4; Peltzer *et al.* 2010:6). Therefore, clinical care directed at individuals living with the virus should include considerations for patient sensitivity to social stigma. Our study revealed that this aspect was lacking in the health centres studies. What has also been found to contribute in minimising stigma is community education toward acceptance of individuals with the condition (Jain *et al*. 2013:5–6). However, approaches in dealing with social stigma ‘must be informed by the prevailing social and cultural forces that provide dominant groups the power to create stigmatizing and discriminatory conditions’ (Mahajan *et al*. 2008:S65). This suggests that it could be effective to conduct continual education of all the women attending ANC at the clinics and address the issue of stigma in particular, whilst maintaining confidentiality.

The matter expressed by participants in that they did not return because of a lack of financial resources and that they were struggling to buy food, suggests that patients’ conditions should be individualised and addressed contextually (McWhinney 1997:136). The challenge of loss to follow-up as a result of socioeconomic factors was also found in a study in Mbarara, Uganda where the top reasons cited for loss to follow-up were lack of transportation or money and work or child care responsibilities (Geng *et al*. 2010:406–410). However, in our study most participants were unemployed and therefore did not cite work responsibilities as a challenge. In South Africa, healthcare practitioners should always raise awareness about available government grants and refer patients to the relevant organisations and departments, such as, for instance, Social Welfare (South African Government 2013).

## Limitations of the study

Since interviews took place on one day by the visiting research assistant, there could have been researcher fatigue which could affect the quality of interaction between the assistant researcher and the participants and which could, in turn, affect collected data quality. Only the women who were from the neighbourhood of the hospital were interviewed for the study – the exclusion of those at distant clinics could have excluded further data on the study phenomenon. However, since the focus of the study was on the depth of information up to the point of information saturation, we believe a wide enough field was covered to address the phenomenon in a fair manner.

## Conclusion

The study has revealed that lack of patient information, poor service rendering, unprofessional conduct, lack of medication, fear of social stigma and poor patient socioeconomic status have a negative effect on patients’ follow-up visits. Adherence by healthcare practitioners to the tenets of professionalism, which entail respect for patients’ rights, confidentiality and communication, could help redress this problem.
